# Decrease of Peripheral and Intestinal NKG2A-Positive T Cells in Patients with Ulcerative Colitis

**DOI:** 10.1371/journal.pone.0044113

**Published:** 2012-09-06

**Authors:** Takehiko Katsurada, Waka Kobayashi, Utano Tomaru, Tomohisa Baba, Shigeru Furukawa, Akihiro Ishizu, Kazuyoshi Takeda, Naoya Sakamoto, Masahiro Asaka, Hiroshi Takeda, Masanori Kasahara

**Affiliations:** 1 Department of Pathology, Hokkaido University Graduate School of Medicine, Sapporo, Japan; 2 Department of Gastroenterology, Hokkaido University Graduate School of Medicine, Sapporo, Japan; 3 Faculty of Health Sciences, Hokkaido University, Sapporo, Japan; 4 Department of Immunology, Juntendo University School of Medicine, Tokyo, Japan; 5 Faculty of Pharmaceutical Sciences, Hokkaido University, Sapporo, Japan; University of Muenster, Germany

## Abstract

To investigate the role of inhibitory natural killer receptors (iNKRs) in inflammatory bowel disease (IBD), we analyzed the expression of NKG2A, one of the iNKRs, on T cells in a mouse colitis model and human IBD. During the active phase of dextran sulfate sodium (DSS)-induced mouse colitis, the frequency of NKG2A+ T cells was significantly decreased in the peripheral blood, and increased in the intestine, suggesting the mobilization of this T cell subset to the sites of inflammation. Administration of anti-NKG2A antibody increased the number of inflammatory foci in DSS-induced colitis, suggesting the involvement of NKG2A+ T cells in this colitis model. In ulcerative colitis (UC) patients, the frequency of peripheral blood NKG2A+ T cells was significantly decreased, compared with Crohn's disease (CD) patients and healthy controls, regardless of clinical conditions such as treatment modalities and disease activity. Notably, in sharp contrast to the DSS-induced mouse colitis model, the frequency of NKG2A+ cells among intestinal T cells was also decreased in UC patients. These results suggest that inadequate local infiltration of NKG2A+ T cells may be involved in the pathogenesis of UC.

## Introduction

The intestinal tract is home to a large number of immune cellular components that continuously encounter abundant exogenous stimuli. Normally, immune responses in the intestine remain in a state of controlled inflammation, mediated by a balance between protective immunity toward pathogens and regulatory mechanisms to circumvent host damages. Inflammatory bowel disease (IBD) is a condition characterized by chronic and refractory intestinal inflammation; there are two distinct, but sometimes overlapping clinical entities that comprise IBD: ulcerative colitis (UC) and Crohn's disease (CD). Although the pathogenesis of IBD remains poorly understood, a large body of evidence indicates that both diseases are caused by imbalances in barrier function and immune responses against pathogens, triggered by infections as well as environmental and genetic factors [1]–[3].

Natural killer (NK) cells are large granular lymphocytes of the innate immune system that produce many cytokines and chemokines, and exert antibody (Ab)-dependent as well as Ab-independent cytotoxicity [4]. NK-cell tolerance to self is ensured in part by the ligation of inhibitory NK receptors (iNKRs) by self-major histocompatibility complex (MHC) class I molecules [5]. These receptors include killer cell immunoglobulin-like receptors and leukocyte immunoglobulin-like receptors in humans, Ly49 molecules in mice, and CD94/NKG2 molecules in both species [6]. All of these receptors are characterized by the presence of an intracytoplasmic immunoreceptor tyrosine-based inhibition motif (ITIM) that is necessary and sufficient for the inhibitory function [7], [8]. Despite being named NK receptors, iNKRs are also expressed on minor subsets of T cells [9]. There is increasing evidence that iNKRs such as NKG2A expressed on T cells are importantly involved in the regulation of immune responses by down-regulating antigen-mediated T-cell effector functions and cytokine release [9]–[11].

Recently, it was reported that intraepithelial CD8+ NKG2A+ γδ+ T cells localized in the small intestine have regulatory potential in celiac disease [12]. However, no studies have addressed the potential role of NKG2A+ T cells in the pathogenesis of IBD. In this study, we examined peripheral blood and intestinal NKG2A+ T cells in a dextran sulfate sodium (DSS)-induced mouse colitis model and UC patients.

## Results

### Frequency of NKG2A+ T Cells in Peripheral Blood Is Decreased in DSS-induced Colitis Mice

**Figure 1:**
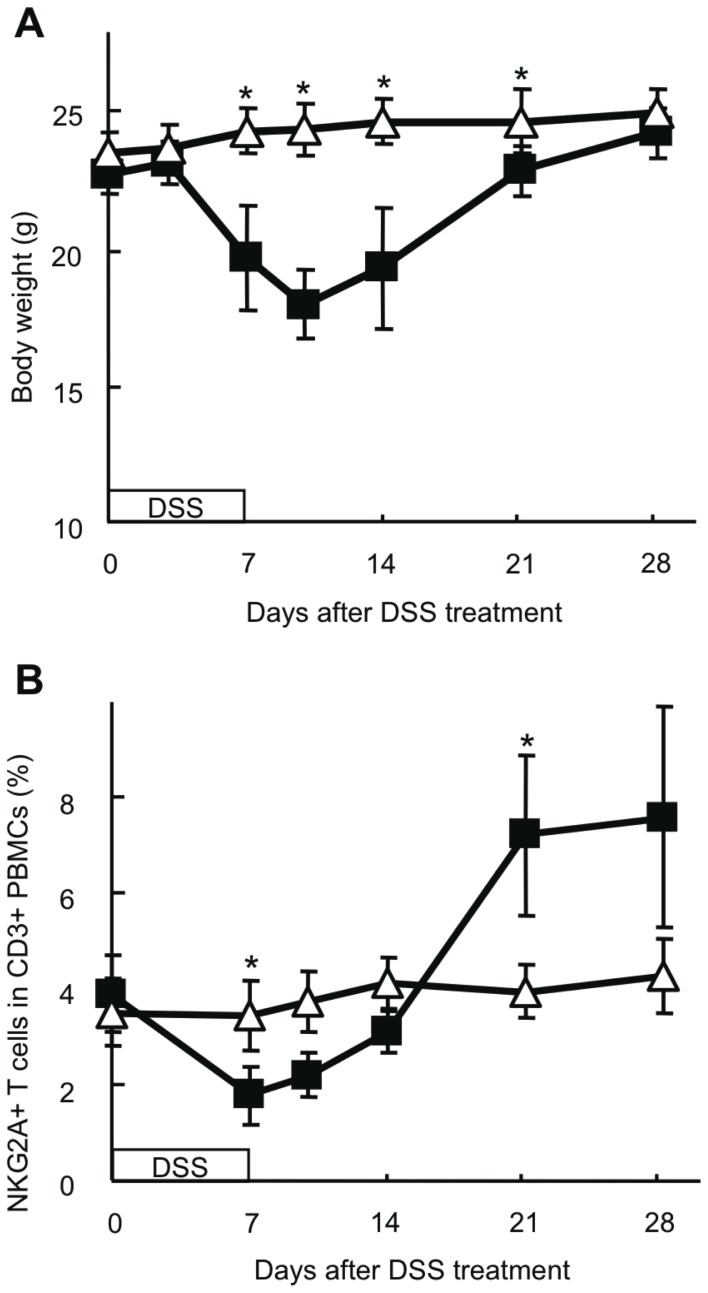
Frequency of NKG2A+ T cells in DSS-induced colitis mice. **A**) Body weight was measured chronologically. **B**) The frequency of NKG2A+ T cells in PBMCs was analyzed by flow cytometry. Data were calculated as the ratio of NKG2A+CD3+ cells to CD3+ cells. ▪, DSS-induced colitis mice; ▵, control mice. For both DSS-induced colitis and control mice, 3 to 10 mice were subjected to analysis at each time point. *P<0.05.

Mice were given 3% DSS in distilled water *ad libitum* for 7 days to induce colitis. On day 3, they started to develop clinical symptoms such as diarrhea, hematochezia, and body weight loss. After discontinuation of DSS treatment, these symptoms were improved around days 10 to 14, and their body weight returned to normal levels around day 21 (Figure 1A). Control mice, which were given distilled water, developed no clinical symptoms at all. We analyzed the frequency of NKG2A+ T cells in the peripheral blood mononuclear cells (PBMCs) of DSS-induced colitis and control mice by flow cytometry (Figure 1B). On day 7, the proportion of NKG2A+ cells among T cells (CD3+ PBMCs) decreased significantly in DSS-treated mice compared with control mice (1.77±0.60% vs 3.45±0.74%, respectively; p = 0.00002). Thereafter, the frequency of NKG2A+ T cells in DSS-treated mice began to increase and returned to the pretreatment levels around day 14. On day 21, when colitis was cured and the body weight was restored to the level equal to that of control mice, the frequency of NKG2A+ T cells in DSS-treated mice exceeded that of control mice (7.22±1.66% vs 3.96±0.5%, respectively; p = 0.00006). Control mice without DSS treatment showed very little change in the frequency of NKG2A+ T cells (3.51±0.71%).

### Frequency of NKG2A+ T Cells Is Increased in Inflamed Intestinal Mucosa

**Figure 2:**
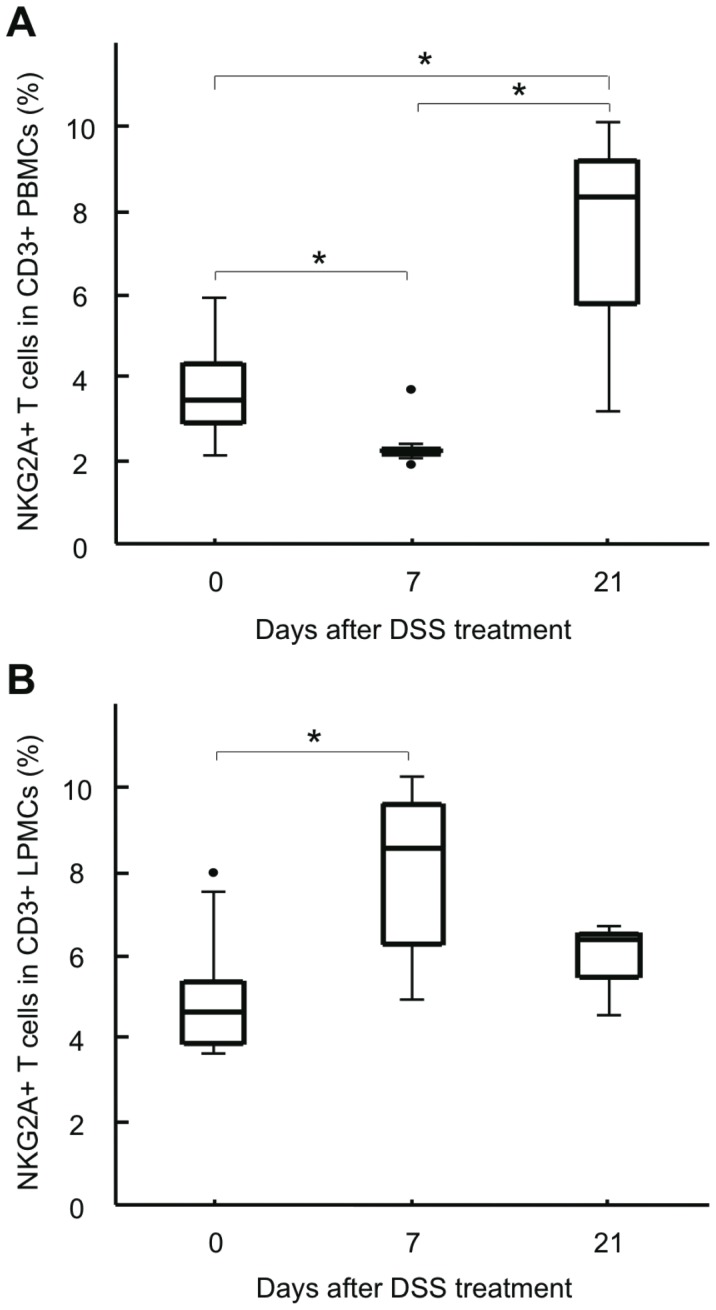
Frequency of NKG2A+ T cells in the peripheral blood and intestine of DSS-induced colitis mice. **A**) Frequency of NKG2A+ T cells in PBMCs of DSS-induced colitis mice. **B**) Frequency of NKG2A+ T cells in LPMCs of DSS-induced colitis mice. Data are expressed as the ratio of NKG2A+CD3+ cells to CD3+ cells. For each time point, 8 or 9 mice were subjected to analysis. *P<0.05.

In addition to NKG2A+ T cells in the peripheral blood, we analyzed the frequency of NKG2A+ T cells in the intestine of DSS-induced colitis mice. Mice were given 5% DSS in distilled water *ad libitum* for 7 days. There is a substantial variability in the effectiveness of different lots of DSS [13]. We therefore chose this concentration of DSS to ensure successful induction of colitis. PBMCs and lamina propria mononuclear cells (LPMCs) were obtained from identical DSS-induced colitis mice on days 0, 7, and 21, and the frequencies of NKG2A+ T cells were measured by flow cytometry. We confirmed that the frequency of NKG2A+ T cells in CD3+ PBMCs was decreased on day 7 and increased on day 21 in DSS-induced colitis mice (3.64±1.20%, 2.39±0.60%, and 7.17±3.57% on days 0, 7, and 21, respectively; Figure 2A). Conversely, the frequency of NKG2A+ T cells in CD3+ LPMCs was markedly increased on day 7 and returned to the level before DSS treatment on day 21 (4.88±1.42%, 7.84±2.12%, and 5.76±1.13% on days 0, 7, and 21, respectively; Figure 2B). These results suggest that, in addition to local expansion, some NKG2A+ T cells were recruited from the peripheral blood to the intestine during active colitis, presumably to suppress local inflammation.

**Figure 3:**
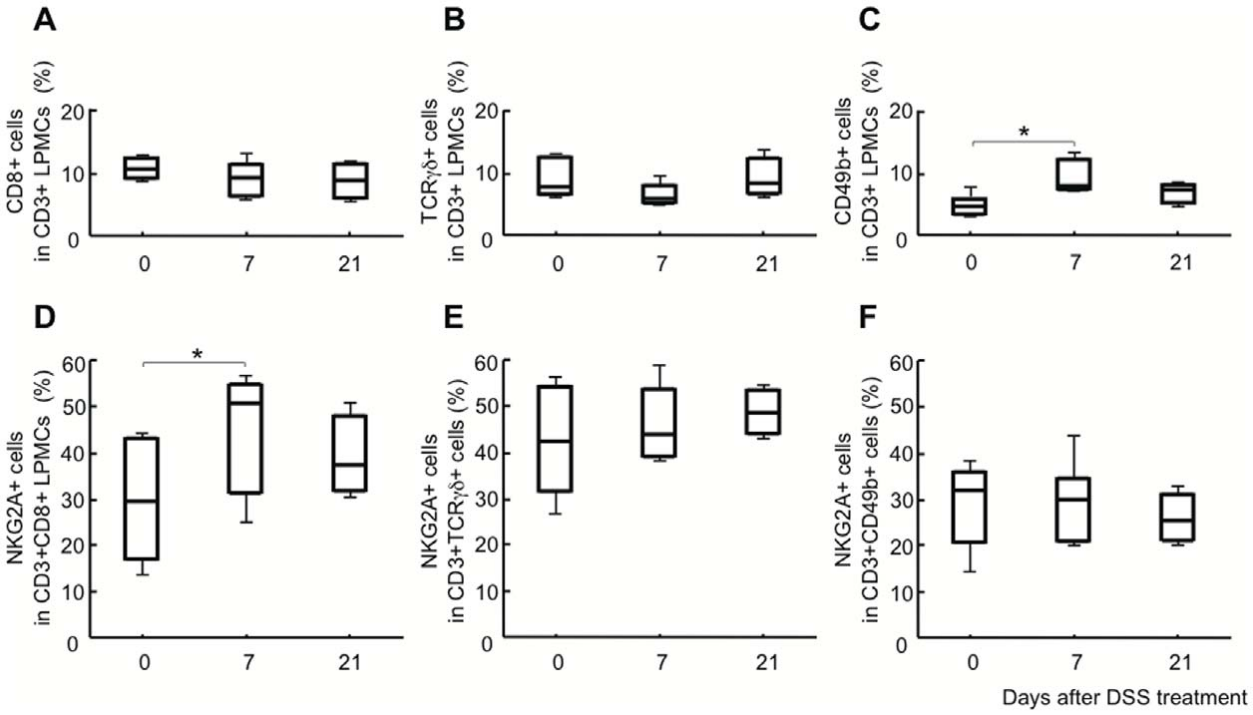
Frequency of NKG2A+ T cells in LPMCs isolated from the intestine of DSS-induced colitis mice. Frequencies of CD8+ T cells in CD3+ LPMCs (A), TCRγδ+ T cells in CD3+ LPMCs (B), CD49b+ T cells in CD3+ LMPCs (C), NKG2A+ cells in CD3+CD8+ LPMCs (D), NKG2A+ cells in CD3+TCRγδ+ LPMCs (E), and NKG2A+ cells in CD3+CD49b+ LPMCs (F) were analyzed by flow cytometry. For each time point, 4 to 7 mice were subjected to analysis. *P<0.05.

Next, we performed flow cytometry analysis to examine NKG2A expression on different T cell subpopulations of LPMCs isolated from DSS-induced colitis mice (Figure 3). Although CD49b expression on T cells was generally lower in LPMCs than in PBMCs (data not shown), the frequency of CD49b+ T cells (panel C) was significantly increased on day 7 in DSS-induced colitis mice (4.49±1.66%, 9.24±2.83%, and 6.71±1.78% on days 0, 7, and 21, respectively). However, the frequency of CD8+ (panel A) or T-cell receptor (TCR) γδ+ (panel B) T cells hovered around 10% (11.22±1.64%, 9.38±2.65%, and 8.98±3.11% on days 0, 7, and 21, respectively, for CD8+ T cells; and 8.84±3.02%, 6.81±1.68%, and 9.09±3.20% on days 0, 7, and 21, respectively, for TCRγδ+ T cells). Interestingly, the frequency of NKG2A+ cells in CD8+ T cells (panel D) was significantly increased on day 7 (29.46±12.69%, 44.59±13.01%, and 39.26±9.02% on days 0, 7, and 21, respectively). There was no significant change in the frequency of NKG2A+ cells in CD3+TCRγδ+ (panel E) or CD3+CD49b+ (panel F) T cells (42.15±11.44%, 46.07±7.93%, and 48.61±5.01% on days 0, 7, and 21, respectively, for CD3+TCRγδ+ T cells; and 28.63±8.96%, 29.44±8.22%, and 25.61±5.28% on days 0, 7, and 21, respectively, for CD3+CD49b+ T cells).

### Treatment with anti-NKG2A Ab Increases the Number of Inflammatory Foci in DSS-induced Colitis

**Figure 4:**
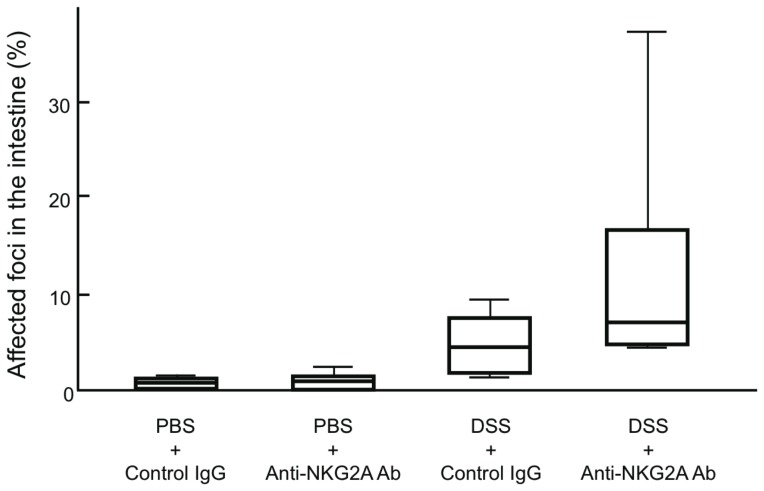
Frequency of inflammatory foci in the intestine of DSS-induced colitis mice treated with anti-NKG2A Ab or control IgG. For Ab blocking experiments, mice were intraperitoneally injected with 300 µg of anti-NKG2A monoclonal Ab or control IgG. The number of inflammatory foci in the intestine was counted histologically on day 6 after DSS administration. Seven mice injected with anti-NKG2D Ab and 8 mice injected with control IgG were subjected to analysis.

To examine whether NKG2A+ T cells exert any immunoregulatory role in DSS-induced colitis, mice were injected intraperitoneally with control IgG or anti-NKG2A Ab and fed 5% DSS in their drinking water. On day 6, mice were sacrificed, and intestinal tissues were subjected to histological analysis. Although the difference fell slightly short of achieving statistical significance, mice injected with anti-NKG2A Ab showed an increased number of inflammatory lesions in the intestine, in comparison with mice injected with control IgG (4.58±3.15% vs 11.83±12.10%, p = 0.06; Figure 4). When mice were fed phosphate-buffered saline (PBS), there was no inflammation in the intestine regardless of whether control IgG or anti-NKG2A Ab was injected.

### Frequency of NKG2A+ T Cells Is Decreased in the PBMCs of UC Patients

**Figure 5:**
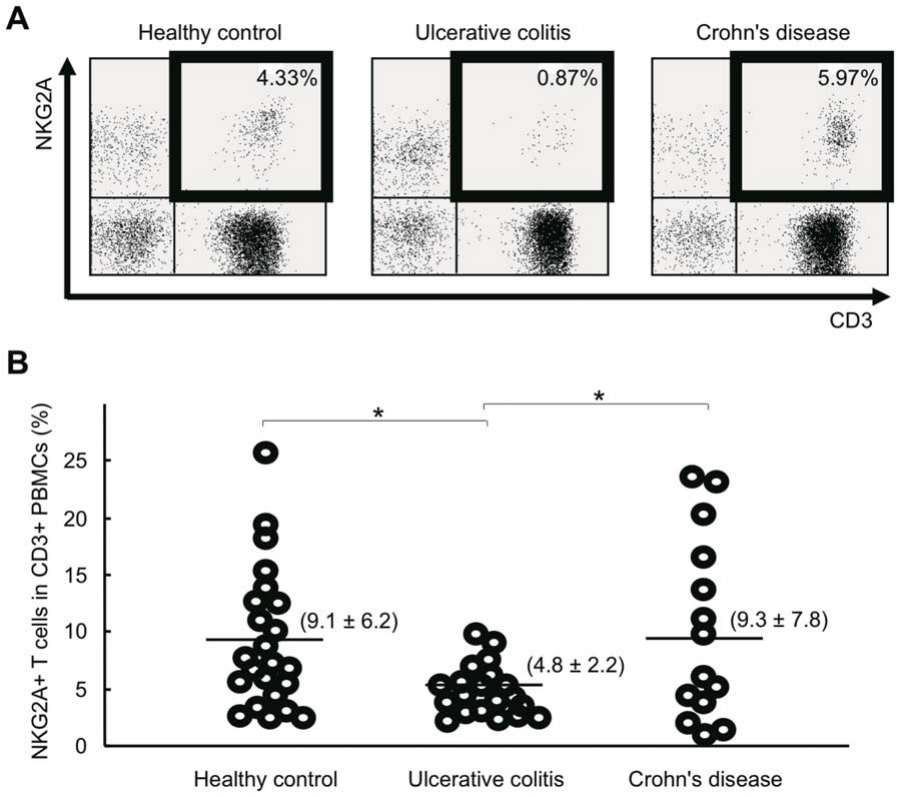
NKG2A+ T cells in the peripheral blood of HCs and IBD patients. **A**) Representative flow cytometry analysis of peripheral blood NKG2A+ T cells in HC and IBD patients. **B**) The ratio of NKG2A+CD3+ cells to CD3+ PBMCs was analyzed by flow cytometry. PBMCs from 23 HCs, 20 patients with UC, and 16 patients with CD were subjected to analysis. Numbers in parentheses represent mean ± standard deviation. *P<0.05.

Because we observed significant changes in the frequency of NKG2A+ T cells in PBMCs of DSS-induced colitis mice, we next examined the frequency of this T cell subset in PBMCs of IBD patients (Figure 5). NKG2A expression on T cells was analyzed by flow cytometry; samples from 20 patients with UC, 16 patients with CD, and 23 HCs were tested (Table 1). We observed a significant decrease of NKG2A+ T cells in the PBMCs of UC patients, compared with HC (p = 0.004) and CD patients (p = 0.019).

**Table 1:** Summary of IBD patients and healthy controls.

	Healthy control	Ulcerative colitis	Crohn's disease
Number of patients	23	20	16
Age range (mean)	26–48 (32.4)	17–48 (31.8)	19–41 (28.6)
Sex:male; female	12; 11	11; 9	8; 8
Type		total colitis; left-sided colitis	small intestine; small and large intestine; large intestine
		13; 7	5; 8; 3
Steroid treatment: +; −		6; 14	NA
Activity: +; −		7; 13	5;11
Colectomy: +; −		5; 15	NA

NA: information not available.

**Figure 6:**
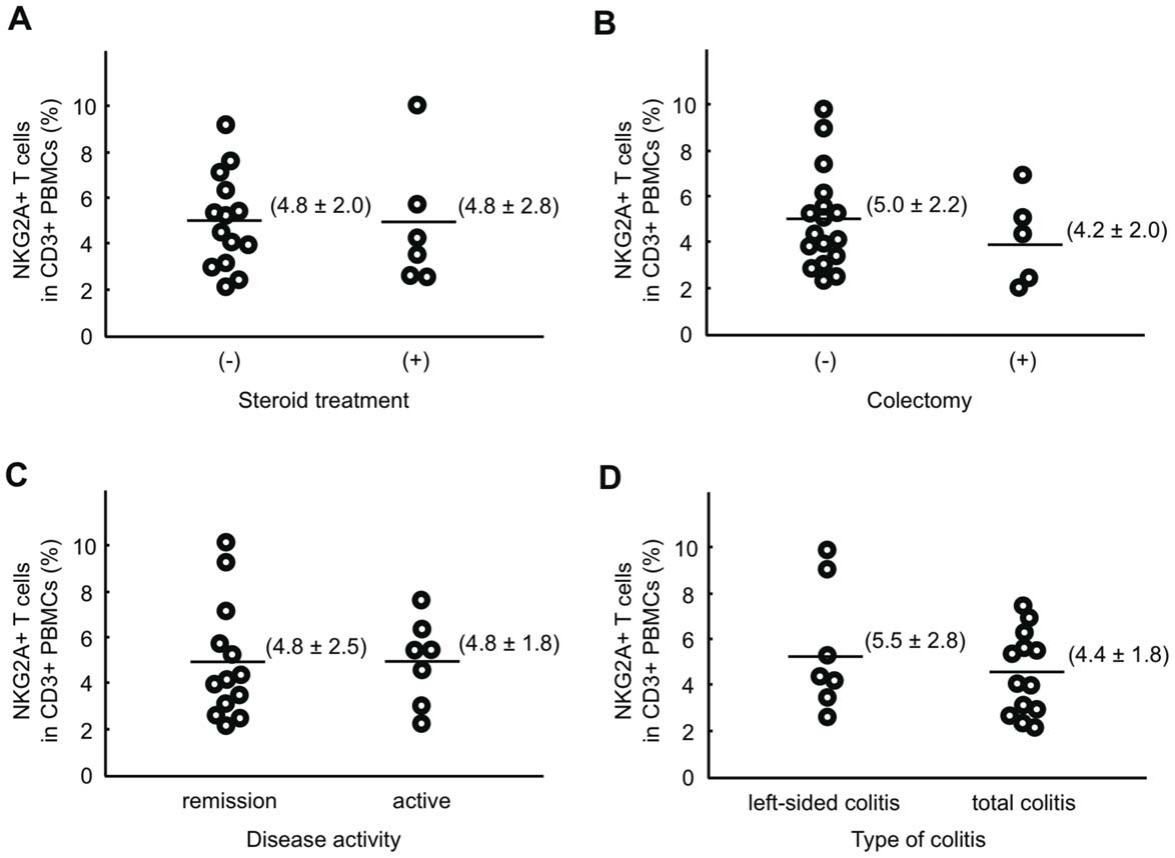
Frequency of NKG2A+ T cells in PBMCs of UC patients subgrouped according to clinical conditions. Data are expressed as the ratio of NKG2A+CD3+ cells to CD3+ cells. Clinical data are summarized in Table 1. Numbers in parentheses represent mean ± standard deviation.

We then examined whether the frequency of NKG2A+ T cells in the PBMCs of UC patients correlated with clinical variables such as the methods of treatment, disease activity, and disease type (Figure 6). The frequency of NKG2A+ T cells was consistently low regardless of whether the patients were treated with steroid (panel A) or received surgical treatment (panel B). Likewise, neither disease activity (panel C) nor the extent of lesions (panel D) affected the frequency of NKG2A+ T cells. We subgrouped CD patients in a similar manner, and examined whether any specific subgroup of patients showed abnormalities in the frequency of peripheral blood NKG2A+ T cells; none of the subgroups showed a decrease in the frequency of peripheral blood NKG2A+ T cells (data not shown).

**Figure 7:**
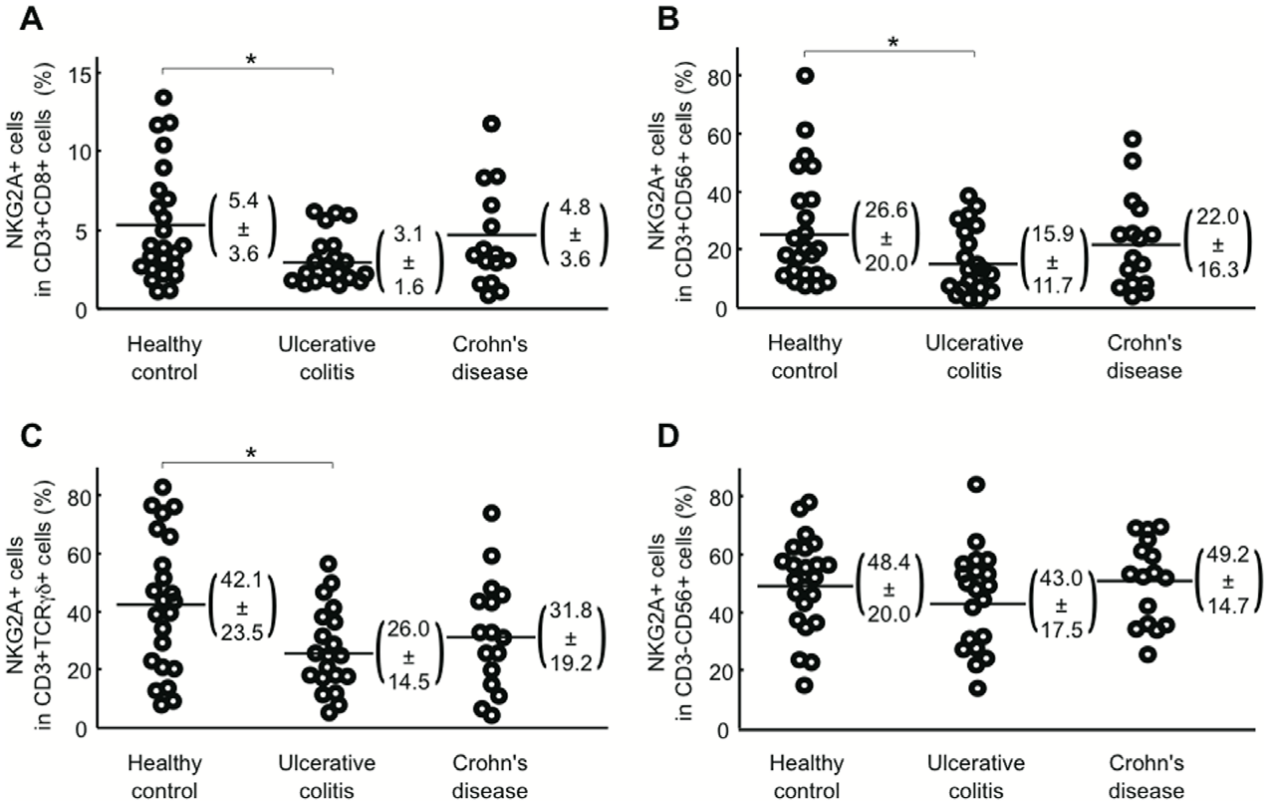
Frequency of NKG2A+ T cells and NKG2A+ NK cells in PBMCs from UC patients, CD patients, and healthy controls. **A**) Ratio of NKG2A+ cells in CD3+CD8+ T cells, **B**) Ratio of NKG2A+ cells in CD3+CD56+ NKT cells, **C**) Ratio of NKG2A+ cells in CD3+TCRγδ+ T cells, and **D**) Ratio of NKG2A+ cells in CD3-CD56+ NK cells. Numbers in parentheses represent mean ± standard deviation. PBMCs isolated from 23 HCs, 20 patients with UC, and 16 patients with CD were subjected to analysis. *P<0.05.

Next, to determine which T cell subsets decreased NKG2A expression in UC patients, we analyzed NKG2A expression on several T cell subsets by flow cytometry (Figure 7). Consistent with a previous observation [14], we did not detect any CD4+ T cells expressing NKG2A in patients or HCs (data not shown). In UC patients, the proportion of NKG2A+ cells was decreased in all T cell subsets including CD3+CD8+ T cells (panel A), CD3+CD56+ NKT cells (panel B), and CD3+TCRγδ+ T cells (panel C). In contrast, the proportion of NKG2A+ cells was not decreased in CD3-CD56+ NK cells (panel D), indicating that the decreased expression of NKG2A in UC patients occurs exclusively in T cells.

### Number of NKG2A+ T Cells Infiltrating the Intestine Is Decreased in UC Patients, in Comparison with CD Patients and HC

**Figure 8:**
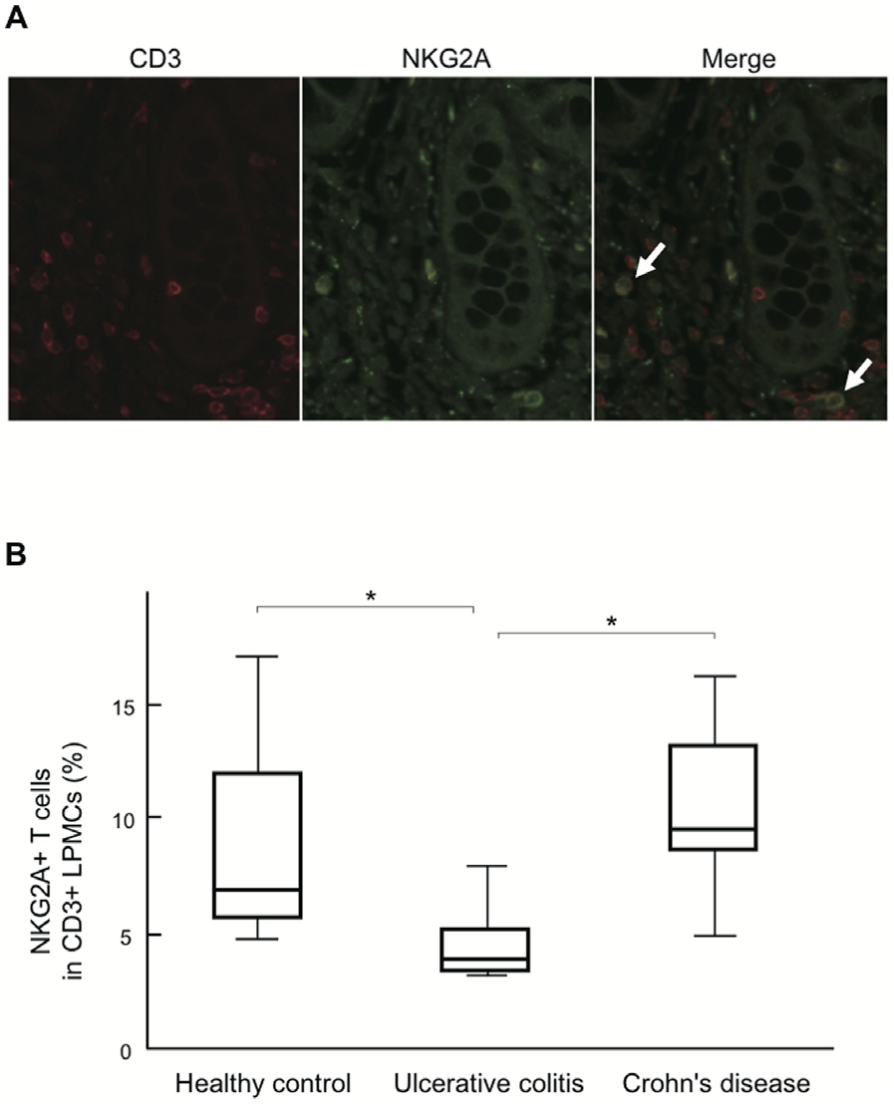
Frequency of NKG2A+ T cells in the intestine of UC patients. **A**) A representative double-stained section of normal intestinal tissues using anti-CD3 and anti-NKG2A Abs. Merged images show double positive cells (arrow). Red, CD3; green, NKG2A. **B**) The frequency of NKG2A+ T cells in the intestine was analyzed by immunohistochemistry. NKG2A+ T cells in the lamina propria were less abundant in UC patients than in HC and CD patients. Tissue sections from 7 HCs, 6 UC patients, and 5 CD patients were subjected to analysis. *P<0.05.

In DSS-induced colitis mice, the frequency of NKG2A+ T cells was decreased in PBMCs, and increased in LPMCs on day 7. To examine whether similar alterations in the frequency of NKG2A+ T cell occurs in UC patients, we analyzed NKG2A+ T cells infiltrating into the affected intestine. Tissue sections derived from the specimens of IBD patients and HCs were double-stained using anti-CD3 and anti-NKG2A Abs. Representative immunofluorescence staining of a HC sample is shown in Figure 8A; CD3+NKG2A+ cells were scattered throughout the lamina propria. CD3+ cells and CD3+NKG2A+ cells were counted in 10 high power fields (HPF) for each section, and the frequency of NKG2A+ T cells in the lamina propria was calculated as a percentage of CD3+NKG2A+ cells among CD3+ cells.

Although massive infiltration of inflammatory cells was observed in the affected mucosa of IBD patients, the number of infiltrating T cells (CD3+ cells) did not differ significantly among UC patients, HC, and CD patients (data not shown). However, in sharp contrast to the mouse model of DSS-induced colitis, the frequency of CD3+NKG2A+ cells in the lamina propria was significantly decreased in UC patients (4.44±1.57%) in comparison with HC and CD patients (8.68±4.73% and 10.49±3.33%, respectively; Figure 8B).

## Discussion

Expression of NKG2A on T cells has been proposed as a mechanism for fine-tuning T cell responses by raising the threshold for TCR triggering, inhibiting cytokine production, and decreasing cytolytic activity [11], [15], [16]. In this study, we demonstrated that NKG2A+ T cells in the intestine underwent dynamic changes during DSS-induced mouse colitis; the frequency of NKG2A+ T cells was significantly increased during the active phase of DSS-induced colitis, and returned to the level before DSS treatment after the inflammation had subsided (Figure 2B). Considering the frequency of NKG2A+ cells in each subpopulation of lamina propria T cells isolated from DSS-induced colitis mice, it is likely that the major population of NKG2A+ T cells in the affected intestine is derived from CD8+ T cells (Figure 3). In the peripheral blood, the frequency of NKG2A+ T cells was decreased during the active phase, but gradually increased after discontinuation of DSS treatment and eventually exceeded that in DSS-untreated mice (Figures 1B and 2A). DSS-induced colitis is a simple model of acute intestinal injury [17]. Thus, the dynamic changes observed in intestinal and peripheral blood NKG2A+ T cells may reflect immune responses mounted against mucosal injuries inflicted by DSS. Although the difference fell slightly short of reaching statistical significance, DSS-fed mice injected with anti-NKG2A Ab showed an increased number of inflammatory foci in the intestine as compared with DSS-fed mice injected with control IgG (Figure 4). In view of the known immunoregulatory function of iNKRs, it seems reasonable to assume that NKG2A+ T cells were recruited to the affected intestine, and expanded there to prevent tissue damages resulting from excessive immune responses.

A growing body of evidence indicates that human NKG2A+ T cells exert immunoregulatory functions in diverse clinical conditions, including infections, autoimmune disorders, and malignant tumors [18]–[21]. Thus, in patients with human T-cell lymphotropic virus 1 (HTLV-1)-associated myelopathy/tropical spastic paraparesis (HAM/TSP), NKG2A+ T cells are significantly decreased, compared with asymptomatic carriers [22]. Saito *et al* [22] showed that a decrease in NKG2A+ CD8+ T cells increased the risk of developing HAM/TSP. Li *et al* [23] demonstrated that patients with systemic lupus erythematosus had lower expression of NKG2A on T cells. On the other hand, increased expression of NKG2A+ on T cells may result in impaired cytotoxic T cell activity in some diseases. In patients with malignant melanoma, increased expression of NKG2A on cytotoxic T cells was shown to impair their lytic activity against melanoma cells [16]. In chronic hepatitis C, the expression of NKG2A+ on NK and CD8+ T cells is increased, presumably acting negatively on the elimination of the virus [21]. These observations indicate that human NKG2A+ T cells have immunoregulatory, in most cases immunosuppressive functions like their mouse counterparts.

The most striking observation made in this study is that the frequency of NKG2A+ T cells was decreased not only in the peripheral blood but also in the inflamed intestine of UC patients (Figures 5 and 8). This is in sharp contrast to the observation made in the DSS-induced mouse model of colitis, where NKG2A+ T cells accumulated in the affected intestine during the active phase of inflammation (Figure 2B). Since iNKR-expressing T cells appear to have immunosuppressive functions, the decreased presence of NKG2A+ T cells in the intestine may result in excessive local inflammation, contributing to the development and persistence of UC.

Regulatory T cells (Treg) have recently received much attention because of their involvement in the maintenance of mucosal microenvironments [24]. In both UC and CD patients, the number of Treg in LPMCs is increased during exacerbation, whereas the frequency of Treg in PBMCs is decreased during exacerbation and increased during remission [25]. In contrast, the frequency of peripheral blood NKG2A+ T cells was consistently low in UC patients regardless of disease activity, disease type, or the methods of treatment (Figure 6). These results suggest that mucosal immunosuppressive functions are predominantly mediated by Treg cells in IBD. A decrease in NKG2A+ T cells may contribute to disease amelioration through a decreased threshold for T cell activation, resulting in decreased cytotoxic activity and cytokine release rather than through decreased immunosuppression. Although the mechanism by which a decrease in NKG2A+ T cells potentially affects susceptibility to UC remains unknown, it must be emphasized that paucity of NKG2A+ T cells was seen in UC, but not CD patients (Figure 5). This observation, together with generally known functions of iNKRs [26], suggests that a decrease in NKG2A+ T cells may be involved in the etiology of UC.

Why is the frequency of NKG2A+ T cells decreased in the peripheral blood and intestinal mucosa of UC patients? One explanation is that UC patients have a decreased total body pool of NKG2A+ T cells. This could happen if expansion of NKG2A+ T cells is impaired or the machinery required to express NKG2A is defective. Interestingly, decreased expression of NKG2A was observed in T, but not NK cells in UC patients (Figure 7). The GATA-3 transcription factor enhances transcription of *NKG2A* in the human NK leukemia cell line NKL, but not in Jurkat T cells [27]. Thus, UC patients might have some mutations in the promoter region of the *NKG2A* gene that affect the binding of transcription factors such as GATA-3. Alternatively, a decrease in the circulating pool of NKG2A+ T cells may occur by an accelerated recruitment of NKG2A+ T cells to the sites of inflammation within the intestine accompanied by an increased local cell death, irreversible down-regulation of NKG2A expression on mobilized T cells, and/or impaired local expansion. However, this explanation appears less likely because the frequency of peripheral blood NKG2A+ T cells remained low in UC patients who underwent a colectomy (Figure 6, panel B). Effective defense against enteric pathogens requires lymphocytes to be appropriately recruited, positioned, and expanded in the intestinal mucosa. This is mediated by a specific repertoire of adhesion molecules, chemokines, and chemokine receptors [28], [29]. A third possibility is that UC patients have some defects in the recruitment of NKG2A+ T cells to the intestine. Our work does not exclude this possibility. However, impaired recruitment alone does not fully explain our observation that the peripheral blood NKG2A+ T cell frequency is consistently low in UC patients.

A significant proportion of healthy individuals have peripheral blood NKG2A+ T cells at frequencies comparable to those in UC patients (Figure 5). This observation may suggest that a decrease in NKG2A+ T cells has a relatively minor pathological role. However, it is also possible that the frequency of peripheral blood NKG2A+ T cells is low in some healthy individuals simply because their T cells are not stimulated to induce NKG2A expression. Unlike healthy individuals, UC patients have elevated serum levels of IL-15 [30]–[32], a cytokine that induces expression of NKG2A on T cells [9]. Nevertheless, UC patients invariably have low frequencies of peripheral blood NKG2A+ T cells (Figures 5 and 6). We therefore examined whether T cells from UC patients and HCs differ in their ability to express NKG2A after IL-15 stimulation, which, however, failed to detect any statistically significant difference between T cells derived from UC patients and HCs (unpublished observation). The possibility still remains open that the response of T cells to other NKG2A-inducing cytokines such as IL-12 [33] and TGF-β [34] is impaired in UC patients.

In summary, our study demonstrates for the first time that the frequency of NKG2A+ T cells is decreased in the peripheral blood and intestinal mucosa of UC patients, implicating this T cell subset as a potential therapeutic target for UC. Further investigation is needed to understand the molecular mechanism by which a decrease in NKG2A+ T cells potentially affects susceptibility to UC.

## Materials and Methods

### Mice

Male BALB/c mice (8–10 weeks old) were purchased from Sankyo Lab., Japan. For induction of experimental colitis, mice were orally administrated 3% or 5% DSS (molecular weight, 40 kDa, Wako) in distilled water for 7 days *ad libitum* [35]. For controls, age-matched BALB/c mice were given distilled water. For the analysis of NKG2A+ T cells in PBMCs and LPMCs, peripheral blood and intestinal tissues were obtained on days 0, 7, 10, 14, 21, and 28 (n = 3 to 10). For blocking experiments using anti-NKG2A Ab, mice were intraperitoneally injected with 300 µg of anti-NKG2A monoclonal Ab or control IgG at three time points: 3 days before, day 0, and day 3 after oral administration of 5% DSS. The anti-NKG2A monoclonal Ab (20d5) was generated as described previously [36]. Control IgG was purchased from Sigma-Aldrich. All animal experiments were done according to the Guide for the Care and Use of Laboratory Animals, Hokkaido University Graduate School of Medicine.

### Patients with IBD and HCs

PBMC samples were obtained from 20 patients with UC, 16 patients with CD, and 23 healthy volunteers. Summarized data for the patients and HCs are given in Table 1. Activity of UC was defined according to the clinical activity index (CAI); patients with index 4 or greater were considered to have active colitis [37]. Activity of CD was defined using the International Organization for the Study of Inflammatory Bowel Disease (IOIBD) score [38]. For immunohistochemical analysis, biopsies and surgical specimens were collected from patients with UC (n = 6) and CD (n = 5). Normal colonic tissues used for immunohistochemical analysis were obtained from surgical specimens of patients with colorectal cancer (n = 7). All patients and healthy volunteers provided written informed consent, and the experiments were approved by the Medical Ethics Committee of Hokkaido University Graduate School of Medicine.

### Cell Isolation

PBMCs were isolated by Ficoll density gradient centrifugation. Isolation of LPMCs from mice with DSS-induced colitis was performed as previously described [39]. Briefly, freshly taken intestinal tissues were washed in PBS and incubated with 1 mmol/L EDTA/PBS for 30 minutes at 37°C. Then, the tissues were minced and digested with type IV collagenase (Sigma-Aldrich) and DNase I (TaKaRa) for 30 minutes in a shaking incubator at 37°C. Released cells were isolated by density gradient centrifugation with Lympholite M (Cedarlane).

### Flow Cytometry

For the detection of NKG2A+ T cells in mouse PBMCs and LPMCs, cells were stained with phycoerythrin (PE)-conjugated rat anti-mouse NKG2A/C/E Ab (clone 20d5, Beckman Coulter) and peridinin chlorophyll-a protein (PerCP)-conjugated hamster anti-mouse CD3 Ab (clone 145-2C11, BD Bioscience). Anti-mouse NKG2A/C/E Ab is operationally monospecific for NKG2A, because CD94/NKG2A complexes, but not other CD94/NKG2 complexes, are expressed on NK cells and T cells [40]. For the analysis of T cell subpopulation, mouse PBMCs and LPMCs were stained with FITC-conjugated anti-CD8 (clone Ly-2, BD Bioscience), FITC-conjugated anti-CD49b (clone HMa2, eBioscience), or FITC-conjugated anti-TCR-γδ (clone 3BioGL3, eBioscience) Ab. Human PBMCs were stained using PE-conjugated anti-NKG2A (clone Z199, Immunotech), FITC-conjugated anti-CD3 (clone UCHT-1, BD Bioscience), RPE-Cy5-conjugated anti-CD3 (clone UCHT-1, DAKO), FITC-conjugated anti-CD8 (clones RPA-T8, BD Bioscience), RPE-Cy5-conjugated anti-CD8 (clone DK25, DAKO), FITC-conjugated anti-CD56 (clone NCAM16.2, BD Bioscience), PE-conjugated anti-CD4 (clone RPA-T4, BD Bioscience), or FITC-conjugated anti-TCRγδ-1 Ab (clone 11F2, BD Bioscience). Isotype controls were used as negative controls. Cells were stained for 30 minutes at 4°C with optimal dilution of Abs. After washing, they were analyzed by flow cytometry (FACSCalibur and CellQuest software, BD Bioscience) by setting a gate around viable lymphocytes based on their forward and side scatter characteristics.

### Histological Analysis and Immunohistochemistry

For histological analysis of DSS-induced colitis mice, formalin-fixed intestinal sections were stained with hematoxylin and eosin (H&E). The frequency of inflammatory foci in the intestine was evaluated through microscopic examination of 60 to 70 tissue sections of comparable size prepared from the whole colon of each mouse. The frequency of inflammatory foci was calculated by dividing the total number of foci by the number of tissue sections analyzed. For immunohistochemical analysis of human intestinal tissues, formalin-fixed tissue sections were deparaffinized in xylene and rehydrated in graded alcohols and distilled water. Deparaffinized and rehydrated slides were processed for antigen retrieval by a standard microwave heating technique. The slides were incubated in PBS with 10% goat serum for 1 hour at room temperature, followed by overnight incubation with goat polyclonal Ab against human NKG2A (1∶100, T-20, Santa Cruz) at 4°C. After incubation, rabbit anti-human CD3 Ab (1∶100, DAKO) was added on slides and incubated for 30 minutes. Negative controls were stained with normal goat serum and rabbit IgG. Sections were labeled with chicken anti-rabbit Alexa 594 and donkey anti-goat Alexa 488 (1∶400, Invitrogen), and examined by fluorescence microscopy. Positive cells in 10 HPFs were counted for each section.

### Statistical Analysis

Data were evaluated using the Student's *t*-test or the Mann-Whitney U-test. P-values less than 0.05 were considered statistically significant.
